# Evaluating the Prevalence and Correlates of Low Resilience in Patients Before Discharge from Acute Mental Health Units in Alberta, Canada

**DOI:** 10.1192/j.eurpsy.2025.390

**Published:** 2025-08-26

**Authors:** E. Owusu, W. Mao, R. Shalaby, H. Elgendy, B. Agyapong, E. Eboreime, M. A. Lawal, N. Nkire, C. T. Hilario, P. Silverstone, P. Chue, X.-M. Lin, Y. Wei, W. Vuong, A. Ohinmaa, V. Taylor, A. J. Greenshaw, V. I. Agyapong

**Affiliations:** 1 Psychiatry, University of Alberta, Edmonton; 2 Psychiatry, Dalhousie University, Halifax; 3 Nursing, University of British Columbia, Okanagan; 4 Addiction and Mental Health, Alberta Health Services; 5 School of Public Health, University of Alberta, Edmonton; 6 Psychiatry, University of Calgary, Calgary, Canada

## Abstract

**Introduction:**

Many people experience at least one traumatic event in their lifetime. Although such traumatic events can precipitate psychiatric disorders, many individuals exhibit high resilience by adapting to such events with little disruption or may recover their baseline level of functioning after a transient symptomatic period.

**Objectives:**

To investigate the prevalence and correlates of low resilience in patients before discharge from psychiatric acute care facilities.

**Methods:**

Respondents for this study were recruited from nine psychiatric in-patient units across Alberta. Demographic and clinical information were collected via a REDCap online survey. The brief resilience scale (BRS) was used to measure low resilience. A chi-square analysis followed by a binary logistic regression model was employed to identify significant predictors of low resilience.

**Results:**

Overall, 1004 participants took part in this study; 360 (35.9%) were less than 25 years old, 269 (34.7%) were above 40 years old, and most participants were females 550 (54.8%) and Caucasians 625 (62.3%). The prevalence of low resilience in this cohort was (555/1004, 55.3%). Respondents who identified as female were one and a half times more likely to show low resilience (OR=1.564; 95% C.I.=1.79-2.10), while individuals with ‘other gender’ identity were three and a half times more likely to evidence low resilience (OR=3.646; 95% C.I.=1.36-9.71) compared to male gender persons. Similarly, Caucasians were two and one-and-a-half times respectively more likely to present with low resilience compared with respondents who identified as Black people (OR=2.21; 95% C.I.=1.45-3.70) and Asians (OR=1.589; 95% C.I.=1.45-2.44). Additionally, persons with a diagnosis of depression were more than two times and four times, respectively, more likely to present with low resilience than those with bipolar disorder (OR=2.567; 95% C.I.=1.72-3.85) and those with schizophrenia (OR=4.081;95% C.I.= 2.63-6.25)

**Conclusions:**

Several demographic and clinical factors were identified as predictors of likely low resilience. The findings may facilitate the identification of vulnerable groups to enable their increased access to support programs that may enhance resilience.

**Disclosure of Interest:**

None Declared

